# Caesarean Section—A Density-Equalizing Mapping Study to Depict Its Global Research Architecture

**DOI:** 10.3390/ijerph121114690

**Published:** 2015-11-17

**Authors:** Dörthe Brüggmann, Lena-Katharina Löhlein, Frank Louwen, David Quarcoo, Jenny Jaque, Doris Klingelhöfer, David A. Groneberg

**Affiliations:** 1 Department of Obstetrics and Gynecology, Keck School of Medicine, University of Southern California, 2020 Zonal Ave, Los Angeles, CA 90033, USA; E-Mails: doerthe.brueggmann@med.usc.edu (D.B.); jenny.jaque@med.usc.edu (J.J.); 2 Department of Female Health and Preventive Medicine, Institute of Occupational Medicine, Social Medicine and Environmental Medicine, Goethe-University, Theodor-Stern-Kai 7, Frankfurt 60590, Germany; E-Mails: arbmed-klinik@uni-frankfurt.de (L.-K.L.); quarcoo@med.uni-frankfurt.de (D.Q.); klingelhoefer@med.uni-frankfurt.de (D.K.); 3 Department of Gynecology and Obstetrics, Goethe-University, Theodor-Stern-Kai 7, Frankfurt 60590, Germany; E-Mail: frank.louwen@kgu.de

**Keywords:** caesarean section, research architecture, scientometrics, density equalizing mapping, gender

## Abstract

Caesarean section (CS) is a common surgical procedure. Although it has been performed in a modern context for about 100 years, there is no concise analysis of the international architecture of caesarean section research output available so far. Therefore, the present study characterizes the global pattern of the related publications by using the NewQIS (New Quality and Quantity Indices in Science) platform, which combines scientometric methods with density equalizing mapping algorithms. The Web of Science was used as a database. 12,608 publications were identified that originated from 131 countries. The leading nations concerning research activity, overall citations and country-specific h-Index were the USA and the United Kingdom. Relation of the research activity to epidemiologic data indicated that Scandinavian countries including Sweden and Finland were leading the field, whereas, in relation to economic data, countries such as Israel and Ireland led. Semi-qualitative indices such as country-specific citation rates ranked Sweden, Norway and Finland in the top positions. International caesarean section research output continues to grow annually in an era where caesarean section rates increased dramatically over the past decades. With regard to increasing employment of scientometric indicators in performance assessment, these findings should provide useful information for those tasked with the improvement of scientific achievements.

## 1. Introduction

Caesarean section (CS) is one of the most common surgical procedures in the field of obstetrics and gynaecology [1]. Almost one in three pregnant women delivers via caesarean section in the United States of America (USA) and Australia; these numbers represent an all time high [2,3]. During the past few decades, the rate of caesarean deliveries has constantly grown in many countries: Declercq *et al.* [4] reported a rise in caesarean section rates between 1987 and 2007 to about 25% in 11 of 21 industrialized countries; Italy, Portugal, the USA and Switzerland were among the nations with the highest numbers [4]. In the USA, the caesarean section rate was stated to be at 32.3% in 2009; it has doubled since 1996 [5]. Unfortunately, these increasing numbers of caesarean deliveries have been mirrored in many other parts of the world. This phenomenon could be seen regardless of social and cultural background or medical and technical determinants of the countries investigated [5]. Hence, the worldwide statistics urged the WHO to issue a recommendation to cut down the global caesarean section rate to a mere 10–15% [6].

Caesarean sections have been described for more than 2000 years. This procedure was named after the Roman emperor Gaius Julius Caesar, although it is very unlikely he was delivered surgically [7]. The maternal mortality of caesarean sections was numbered at 70%–100% in the 19th century, which improved after Ignaz Semmelweis discovered asepsis as the cornerstone of every surgical procedure [8]. During the last 100 years, safety and access to caesarean sections have even more increased leading to an overall improvement of maternal and fetal outcomes—Particularly in high-income countries [9]. While the wide adoption of caesarean sections has advanced the obstetrical care drastically, it becomes evident that the most recent rise in procedure numbers does not translate in even better outcomes for mother and child [9]. On the contrary, the high caesarean section rate might contribute to perinatal morbidity and mortality since these procedures are frequently associated with adverse outcomes. Intraoperative complications include excessive blood loss or injury to adjacent organs. Infections, the most frequent morbidity associated with caesarean sections, wound hematoma, severe anemia or venous thrombembolism can be experienced in the postpartum period [8]. Additionally, a history of caesarean section might affect the course of subsequent pregnancies: Placenta previa and accreta pose the most significant risk to the patient’s health, they may lead to life-threatening hemorrhage and hysterectomy [8,9]. A Danish cohort study described increased risks of stillbirth and ectopic pregnancy following caesarean sections. Their data on miscarriage were inconsistent or revealed no increased risk [10,11]. Also, birth by caesarean section might impact the development of the newborn since the procedure was linked to an increased risk of immune conditions (e.g., systemic connective tissue disorders, juvenile arthritis, inflammatory bowel disease and gastroenteritis) [12,13]. An elevated risk for childhood asthma was reported for children delivered by caesarean section, although sibling analysis showed no causal relationship [12,13,14]. In addition, birth by caesarean section might be associated with a 20% increased likelihood to be diagnosed for Autism Spectrum Disorders [15]. The data regarding type 1 diabetes remain equivocal: While a cohort of nearly 3 million Swedish children revealed a small—But not causal—Association between elective caesarean section and type 1 diabetes, this result could not be reported by other groups [16,17,18].

In light of these findings, there is a need for an ongoing debate regarding the question of the favorable caesarean section rate in a population. Discussions should include clinical, cultural and psychological issues and also touch areas of public health and health economics. It is important to strengthen research and to ultimately find ways to decrease the current caesarean section rates by defining guidelines and re-evaluating indications critically. In contrast to the dimension of caesarean section-related research, only little is known about the global research architecture and existing collaborative networks. Therefore, we here conducted the first detailed density-equalizing mapping study that integrates scientometric data in order to draw a sketch of the global caesarean section research landscape over the past 100 years.

## 2. Experimental Section

### 2.1. NewQIS Platform

We used the New Quality and Quantity Indices in Science (NewQIS) initiative’s platform to assess the worldwide activities in the field of caesarean section research [19,20]. The platform combines scientometrics with advanced visualization techniques such as density equalizing calculations [21] to visualize research activity in quantitative and semi-qualitative terms [19,20]. The methods were previously established and used in numerous NewQIS projects in the areas of public health, health policy research [22,23,24,25,26], or internal medicine [27,28].

### 2.2. Data Source

To retrieve publications, we used the database Web of Science (WoS) from Thomson Reuters as described in previous NewQIS studies [29,30]. WoS was selected as data source because it provides a broad range of bibliographic data attached to the listed publications such as country of origin or subject categories, and supplies the Citation Report, a WoS specific feature to extract citation information.

### 2.3. Search Strategy

The search-term (cesarean * OR caesarean * OR abdominal *) AND (section * OR deliver *) OR (c-section *) was inserted into the WoS search field to approximate the overall number of published items referring to caesarean section.

### 2.4. Timeframe

The timeframe of analyzed caesarean section research covered the time span between 1900 (1 January) and 2013 (31 December). As in previous studies, results from 2014 and 2015 were not considered due to incomplete data acquisition at the time the study was performed (*i.e.*, citation rate).

### 2.5. Data Analysis and Categorization

We downloaded and saved all publications identified by our search term in a Plain text format using the download tool provided by the WoS. We created an interim database by collecting all metadata related to the items. With the help of these metadata, publications were categorized with respect to country of origin, publication date, source title, and authors. As semi-qualitative measures, the numbers of citations were retrieved for each publication and the average citations per item (citation rate) were calculated.

After transfer of the raw data to excel charts, the findings were illustrated in diagrams and visualized by density-equalizing mapping projections (DEMP). The presently calculated DEMP are based on the algorithm by Gastner and Newman [21] that allows for resizing the country areas and the lengths of their borders based on particular mathematical factors. Hence, in this DEMP approach, the territories of the different countries, which published caesarean section-related research, were resized in proportion to the analyzed variable (e.g., total number of publications and citations, citation rate and h-Index). By this process, we created an expressive global sketch of caesarean section research activities representing the worldwide distribution of country-specific publication numbers and the country-specific average citation rates [21]. Regression analysis was used to investigate the timely evolution of caesarean section research. We calculated the coefficient of determination (r^2^) that represented the slope of the growth in scientific output. Based on r^2^, we quantified the increase in number of cesarean section-related articles in correlation to two time periods, e.g., 1900–2012, which includes the entire time period of our investigation, *versus* 1970–2012, representing the time period where a steady and steep publication increase was visible.

### 2.6. Research Activity in Relation to Epidemiologic Data

Caesarean section publication output was related to epidemiologic figures in order to assess the magnitude of a country’s research activity in this specific field. It was hypothesized that a country with high numbers of caesarean sections should also conduct and publish more research in this area of obstetrics. Epidemiologic data was obtained from a World Health Report 2010 [31].

### 2.7. Economic Analysis of Caesarean Section Research Activity

Quantitative output figures were related to economic data provided by the World Bank (www.worldbank.org) in order to analyze country-specific research activities in relation to the financial abilities of single countries to invest in caesarean section-related research. We stratified investigated countries into the following groups according to their Gross National Income *per capita* (GNI) in 2014: High-income, upper-middle-income, lower-middle-income and low-income countries. This assessment is based on the classification provided by the World Bank (http://data.worldbank.org/country/).

### 2.8. Analysis of Collaborations

To investigate research collaborations between the different countries, the affiliations of the publications were analyzed as previously described for other diseases [29,32]. In brief, if at least two authors coming from two different countries contributed to a caesarean section-related publication, this relationship was defined as one collaborative publication. To visualize the collaborative productivity for each pair of countries, a vector was calculated. The line width and grey shade of the vector were proportional to the number of identified caesarean section cooperations [29,32].

## 3. Results and Discussion

### 3.1. Global Caesarean Section Research Activity

**Figure 1:**
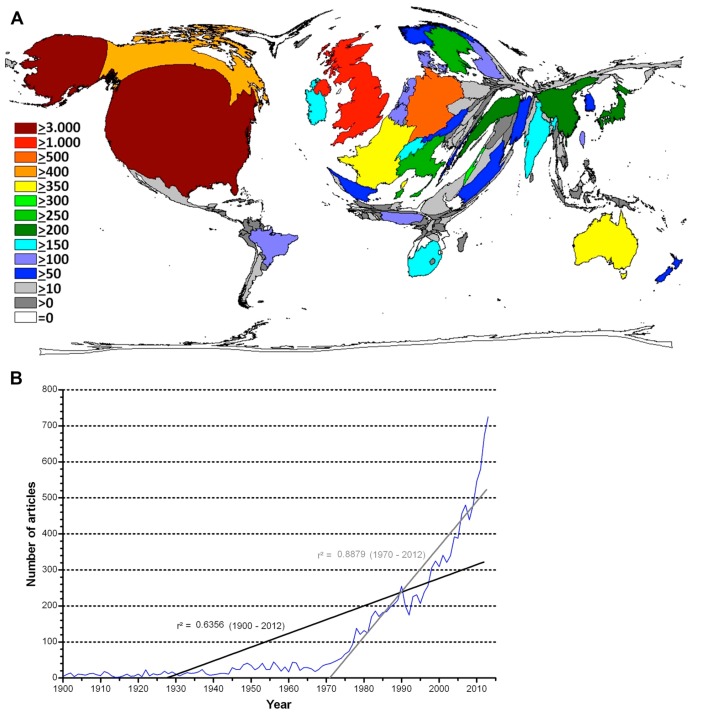
Publication output. (**A**) Global density-equalizing map of total caesarean section publications over the past 113 years. The area of each country was scaled in proportion to its total number of publications. Colours encode numbers of published items. (**B**) Regression analysis of total caesarean section-related publications between 1900 and 2012.

In total, 12,608 publications related to the term “caesarean section” were identified (92.8% in English). These were published by scientists from 131 nations. The USA was the most productive country with a total of 3679 caesarean section-related publications (29%), followed by the United Kingdom (UK) with a total of 1712 publications (14%). Germany ranked third with 506 publications (4%) and was followed by Canada (491 publications, 3.8%), Australia, France, Israel, Italy, Sweden and Turkey. The latter ranged between 221 and 373 publications. The DEMP calculations exemplify (Figure 1A) that the USA has the leading global role in caesarean section research followed by Western Europe. Major parts of Asia including Russia, Africa and South America occupy only a minimal area on the map.

Based on the value of r^2^, which represented the slope of the increase in scientific output, we analyzed the timely evolution of caesarean section research. It showed a value of r^2^ = 0.6356 for the period from 1900 to 2012. For the period between 1970 and 2012, r^2^ = 0.8879 was computed, which indicated a steep increase in the caesarean section research activities since 1970 (Figure 1B).

### 3.2. Research Activity in Relation to Caesarean Section Epidemiologic Data

Epidemiologic estimates indicate a large variety in absolute caesarean section numbers between countries. Hence, we related the total number of caesarean section-related articles, which were published in a particular country, to the numbers of caesarean sections performed per year. In this analysis, we included only countries that published at least 50 caesarean section-related articles in the analyzed timeframe. 

The two Scandinavian countries, Sweden (18,510 caesarean sections per year) and Finland (9620 caesarean sections per year), led the top-ranking countries with 13.56 and 13.00 caesarean section-related publications per 1000 caesarean section cases. The UK (163,460 caesarean sections per year) was ranked 5th with 10.47 publications per 1000 annual caesarean sections. Norway (9630 caesarean section per year) and Denmark (13,270 caesarean sections per year) were ranked 6th and 7th, with 9.66 and 8.74 publications per 1000 cases, respectively. For the USA (1,332,900 caesarean sections per year), we documented a rate of 2.76 caesarean sections-related publications per 1000 caesarean sections. The USA ranked 15th among all analyzed countries. Countries with higher total numbers of caesarean sections including Brazil with 1,425,200 caesarean sections per year, India with 2,287,610 caesarean sections per year and China with 4,696,710 caesarean sections per year were ranked 27th to 29th with 0.09, 0.08 and 0.05 caesarean section-related publications per 1000 caesarean sections cases, respectively. In this analysis, the 19 top-ranked countries were part of the high-income group (as defined by the World Bank). They showed a wide range regarding their publication activity per annual caesarean sections performed with 13.56 publications per 1000 annual caesarean sections in Sweden *versus* 1.22 issued publications per 1000 caesarean sections in Japan. As representatives of upper-middle-income countries, we identified South Africa, Turkey, Iran, Brazil, and China. The only two low-income countries we could identify in this analysis were Nigeria and India (Table 1).

### 3.3. Economic Analysis of Caesarean Section Research Activity

The number of caesarean section publications was related to the absolute Gross Domestic Product in US Dollars (GDP * 100,000,000 current USD) to analyze the economic feasibility of a country to invest in research with regard to its absolute financial power (Table 2). Here, Israel was ranked first with 107.73 publications per GDP * 100,000,000 current USD. It was followed by Ireland and the UK with 82.3 and 63.92 publications per GDP * 100,000,000 current USD, respectively (Table 2). In the top ten ranking, three Scandinavian countries were present: Finland was at position 5, Sweden at position 6 and Denmark at position 7 (46.76, 43.3 and 34.54 caesarean section-related publications/GDP * 100,000,000 current USD, respectively). The USA was ranked 15th with 21.94 caesarean section-related publications/GDP * 100,000,000 current USD. Although they also have a high financial power, Japan or China were ranked last, and can be found at position 28 and 29 with 4.47 and 2.35 caesarean section-related publications per GDP * 100,000,000 current USD, respectively. When number of publications on caesarean sections was related to GDP, five upper-middle-income countries were ranked in position 4 (South Africa), 9 (Turkey), 14 (Iran), 26 (Brazil) and 29 (China). The two representatives of the lower-middle income countries Nigeria and India were found in positions 16 and 23 (Table 2).

**Table 1:** Country specific ranking of caesarean section (CS) related publications per total annual CS cases. Cut off level 50 CS related publications.

	Country	Number of CS (in Thousands)	Number of Publications	Number of Publications/Number of CS (per 1000 CS)	Type of Country Based on GNI
1.	Sweden	18.51	251	13.56	HIG
2.	Finland	9.62	125	13.00	HIG
3.	Israel	26.74	313	11.71	HIG
4.	Ireland	18.08	191	10.57	HIG
5.	United Kingdom	163.46	1712	10.47	HIG
6.	Norway	9.63	93	9.66	HIG
7.	Denmark	13.27	116	8.74	HIG
8.	Switzerland	21.10	155	7.35	HIG
9.	Belgium	18.92	120	6.34	HIG
10.	Netherlands	24.98	136	5.45	HIG
11.	Canada	92.84	491	5.29	HIG
12.	New Zealand	11.83	59	4.99	HIG
13.	Australia	80.90	373	4.61	HIG
14.	Austria	20.60	82	3.98	HIG
15.	United States	1332.90	3679	2.76	HIG
16.	Germany	185.15	506	2.73	HIG
17.	France	141.38	352	2.49	HIG
18.	Italy	208.57	292	1.40	HIG
19.	Japan	179.92	220	1.22	HIG
20.	Nigeria	108.50	101	0.93	LMIG
21.	Saudi Arabia	76.83	66	0.86	HIG
22.	South Africa	224.75	189	0.84	UMIG
23.	Turkey	285.78	221	0.77	UMIG
24.	Spain	127.17	74	0.58	HIG
25.	Republic of Korea	170.40	86	0.50	HIG
26.	Iran	581.57	83	0.14	UMIG
27.	Brazil	1425.20	132	0.09	UMIG
28.	India	2287.61	176	0.08	LMIG
29.	China	4696.71	217	0.05	UMIG

HIG: High-income group (white), UMIG: Upper-Middle-Income group (light grey), LMIG: Lower-Middle-Income group (grey)

**Table 2:** Ratio of the number of CS publications and Gross Domestic Product (GDP) in current US-Dollars (USD).

	Country	Number of Publications	GDP Current USD	Number of Publications/GDP * 100,000,000 Current USD	Type of Country Based on GNI
1.	Israel	313	2.90551E+11	107.7265027	HIG
2.	Ireland	191	2.32077E+11	82.30012236	HIG
3.	United Kingdom	1712	2.67845E+12	63.91744765	HIG
4.	South Africa	189	3.66058E+11	51.63117449	UMIG
5.	Finland	125	2.67329E+11	46.75893024	HIG
6.	Sweden	251	5.7968E+11	43.29975268	HIG
7.	Denmark	116	3.35878E+11	34.53639595	HIG
8.	New Zealand	59	1.85788E+11	31.75665583	HIG
9.	Turkey	221	8.22135E+11	26.88122398	UMIG
10.	Canada	491	1.82677E+12	26.87806272	HIG
11.	Australia	373	1.56037E+12	23.90454884	HIG
12.	Belgium	120	5.24806E+11	22.86561292	HIG
13.	Switzerland	155	6.85434E+11	22.61340379	HIG
14.	Iran	83	3.68904E+11	22.49905691	UMIG
15.	United States	3679	1.67681E+13	21.9404703	HIG
16.	Nigeria	101	5.21803E+11	19.35595217	LMIG
17.	Austria	82	4.28322E+11	19.14447999	HIG
18.	Norway	93	5.1258E+11	18.14349424	HIG
19.	Netherlands	136	8.53539E+11	15.93365317	HIG
20.	Italy	292	2.14948E+12	13.5846524	HIG
21.	Germany	506	3.73026E+12	13.56473604	HIG
22.	France	352	2.80643E+12	12.54263436	HIG
23.	India	176	1.87514E+12	9.38595843	LMIG
24.	Saudi Arabia	66	7.4845E+11	8.818229043	HIG
25.	Republic of Korea	86	1.30455E+12	6.592291451	HIG
26.	Brazil	132	2.24567E+12	5.877970573	UMIG
27.	Spain	74	1.39304E+12	5.312122451	HIG
28.	Japan	220	4.91956E+12	4.47194182	HIG
29.	China	217	9.24027E+12	2.348416111	UMIG

HIG: High-income group (white), UMIG: Upper-Middle-Income group (light grey), LMIG: Lower-Middle-Income group (grey)

To evaluate the magnitude of research activity in high-income countries in regards to their financial power *per capita*, we calculated the number of caesarean section-related publications/GDP *per capita* current 1000 USD (Table 3). We found that the USA led this ranking with 69.36 caesarean section publications/GDP *per capita* in current 1000 USD, followed by the UK with 40.98 and Germany with 10.94 publications/GDP *per capita* in current 1000 USD, respectively. Scandinavian countries were ranked at position 10 (Sweden), 15 (Finland), 18 (Denmark) and 22 (Norway) with 4.16, 2.54, 1.94 and 0.92 publications/GDP *per capita* in current 1000 USD, respectively (Table 3).

**Table 3:** Number of caesarean section publications/GDP *per capita* current 1000 USD in high-income countries.

	Country	Number of Publications	GDP *per Capita* Current 1000 USD	Number of Publications/ GDP *per Capita* Current 1000 USD
1.	United States	3679	53.042	69.36012971
2.	United Kingdom	1712	41.7811	40.97546498
3.	Germany	506	46.2514	10.94020938
4.	Canada	491	51.9643	9.448794653
5.	Israel	313	36.0507	8.682216989
6.	France	352	42.5604	8.270598961
7.	Italy	292	35.6856	8.182572242
8.	Japan	220	38.6337	5.694510233
9.	Australia	373	67.463	5.528956613
10.	Sweden	251	60.3809	4.156943669
11.	Ireland	191	50.4784	3.783796634
12.	Republic of Korea	86	25.977	3.310620934
13.	Netherlands	136	50.7925	2.677560663
14.	Belgium	120	46.9296	2.557021581
15.	Finland	125	49.1506	2.543203949
16.	Saudi Arabia	66	25.9618	2.542196612
17.	Spain	74	29.8821	2.476398914
18.	Denmark	116	59.8186	1.93919617
19.	Switzerland	155	84.7484	1.828943083
20.	Austria	82	50.5107	1.623418404
21.	New Zealand	59	41.8243	1.410663179
22.	Norway	93	100.8984	0.921719274

### 3.4. Citation Analysis

**Figure 2:**
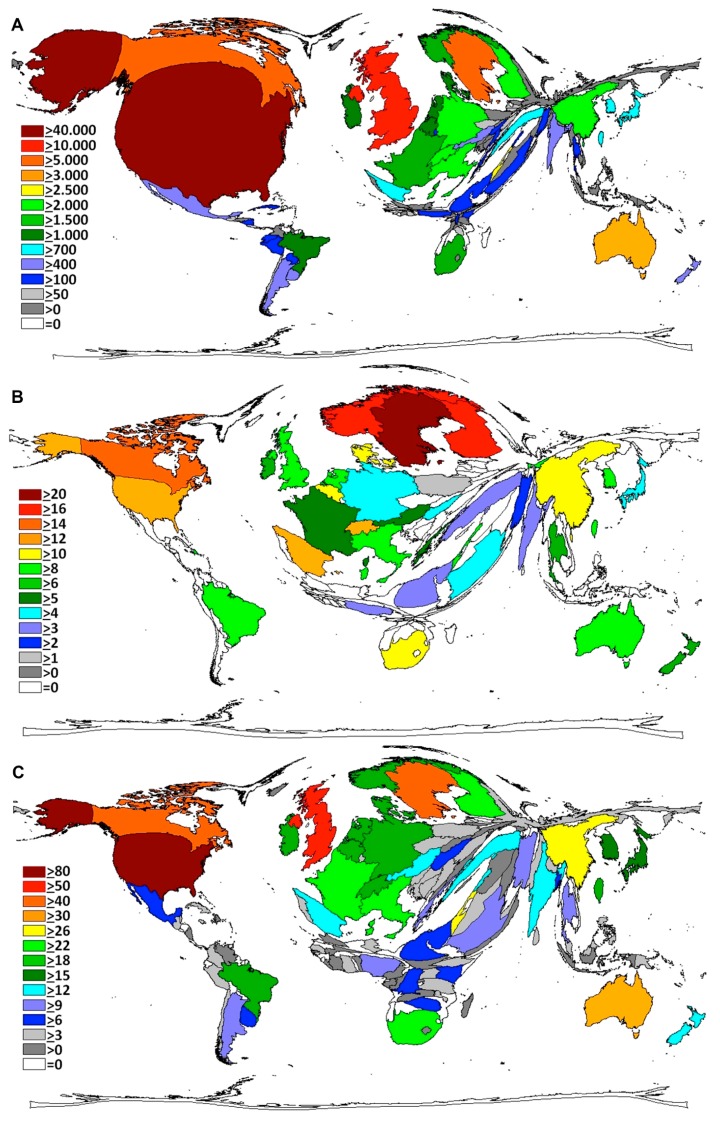
Density-equalizing Map Projections. (**A**) Country specific number of citations of caesarean section publications. The area of each country was scaled in proportion to its total number of citations. Colours encode numbers of citations. (**B**) Country specific citation rate (Citations per publication). The area of each country was scaled in proportion to the citation rate levels. (**C**) Country specific h-Index. Colours encode h-Index level.

The total citation analysis (Figure 2A) parallels the results of the global publication activity. Publications coming out of the USA received the highest number of citations with 46,194 references followed by the publications from the UK receiving 15,377 citations. Canada is ranked 3rd with 7423 citations followed by Sweden (5113), Australia (3483), Israel (2547), China (2438), Italy (2360), Switzerland (2101), Finland (2044) and Germany (2027). DEMP calculations for citation activity exhibit a similar pattern to the total publications DEMP analysis with some minor exceptions, *i.e.*, Sweden occupied a bigger area and Germany a smaller (Figure 2A).

The country-specific citation rate (Figure 2B) was used as a semi-qualitative benchmark (citations per CS publication of a country). It demonstrated that Sweden had the highest citation rate with 20.4 citations per caesarean section publication. The two Scandinavian countries Norway and Finland were placed second with citation rates of 16.3 each, followed by Canada (15.1) and Switzerland with 13.6 citations per caesarean section publication. The USA has a citation rate of 12.6 citations per caesarean section publication. The DEMP shows a distorted global map with the territories of the Scandinavian countries being increased to a maximum.

When the country-specific h-Index (hI) for caesarean section-related publications was analysed (Figure 2C), the USA ranked first with 84 publications that were at least cited 84 times. It was followed by the UK with an h-Index of 52, then Canada (hI = 41) and Sweden (hI = 40). Taiwan with a total number of 109 publications had a rather high h-index of 27. We found h-indices between 20 and 24 for European countries such as Germany, France and Italy.

### 3.5. International Collaboration Analysis

**Figure 3:**
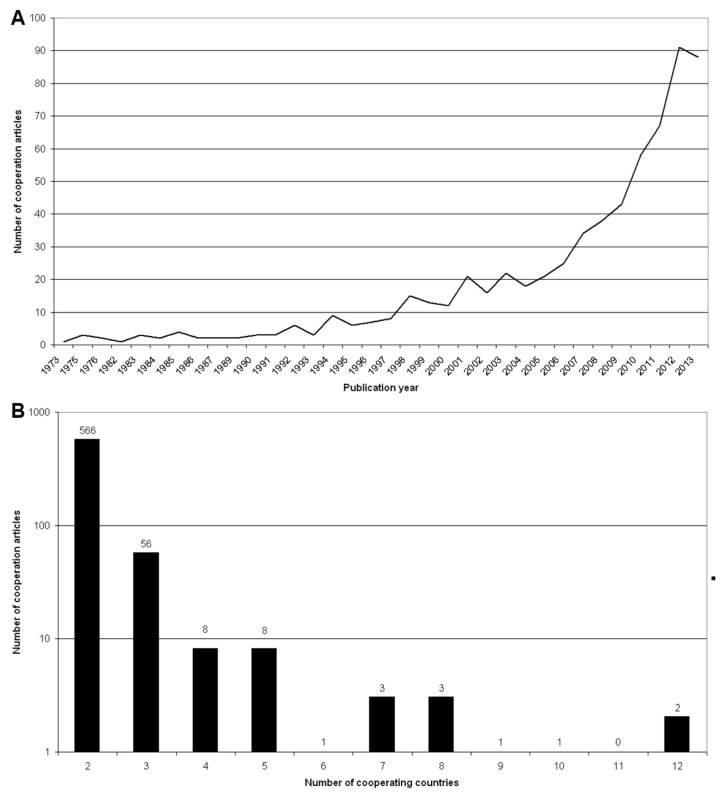
International collaborations. (**A**) Evolution since 1973. (**B**) Numbers of collaborating countries (logarithmic).

Matrix analysis revealed that among the 12,608 publications, only 1237 publications were a result of international cooperations between two or more countries. Since 2005, the number of relevant collaborations increased steeply (Figure 3A). Bilateral cooperations were the most common collaboration type leading to a total of 561 publications. They were followed by trilateral cooperations (56 publications). We documented collaborations between four countries in only eight publications, five country cooperations in eight publications (Figure 3B).

**Figure 4:**
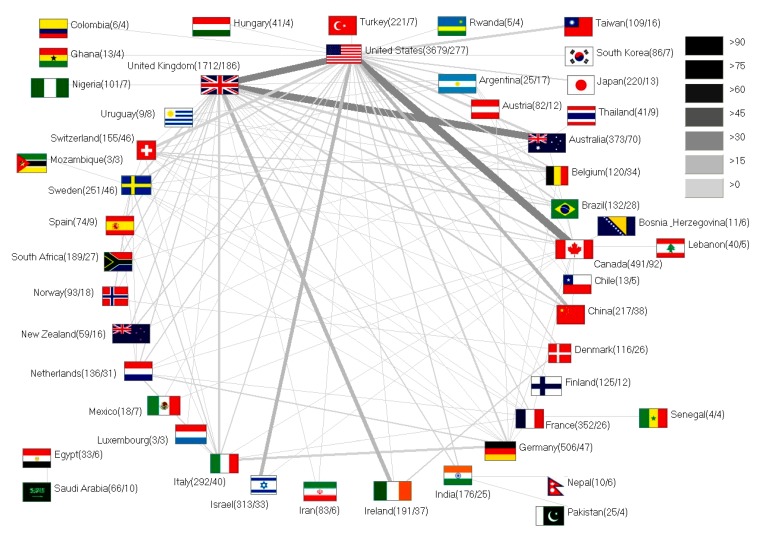
Net diagram of international cooperations. Line width and grey scale encode numbers of collaborations. Numbers in brackets (number of publications of a specific country/number of collaboration articles of a specific country).

A net chart exemplifies the magnitude of collaborations between the different countries (Figure 4). Once again, the USA dominated with 277 collaborative articles. The most common cooperation was one between the USA and Canada having authored 41 joint publications, followed by USA with the UK leading to 33 publications.

### 3.6. Subject Area Analysis

**Figure 5:**
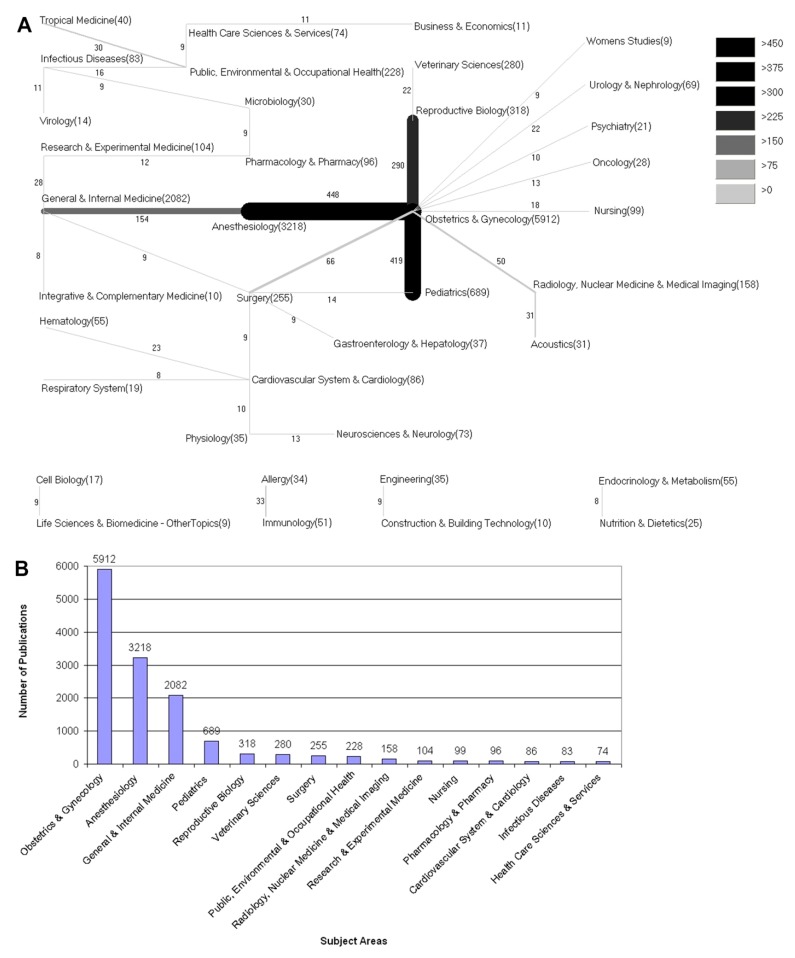
Subject areas. (**A**) Combination of the most assigned subject areas (**B**) Number of publications regarding the most assigned subject areas.

The most frequently assigned subject category was Obstetrics and Gynecology with 5912 assigned publications (Figure 5). It was followed by Anesthesiology (3218) and Pediatrics (689). The three most frequent combined subject areas were Obstetrics and Gynecology with Anesthesiology (448), Obstetrics and Gynecology with Pediatrics (689) and Obstetrics and Gynecology with Reproductive Biology (293).

### 3.7. Discussion

In view of the increasing incidence of caesarean sections over the past decades, the “New Quality and Quantity Indices in Science” (NewQIS) initiative [19,20] decided to assess this common surgical procedure using an in depth study protocol, which combines modern visualization techniques such as density equalizing mapping [21] with scientometric tools. In this respect, our study displays the first detailed analysis of the global caesarean section research architecture. Our findings demonstrate that the USA produced the most caesarean section-related publications in the investigated timeframe, which also received the highest number of citations and led to the highest country-specific h-Index. Further, the USA was involved in the most research collaborations working on this topic. When research activity was related to epidemiologic data such as numbers of annually performed caesarean sections or to economic resources, this picture changed: We found the highest research activity related to 1000 caesarean sections per year in Scandinavian countries such as Sweden and Finland. Overall, high-income countries dominated when research activity was assessed in regards to economic resources, e.g., Israel and Ireland published the most research in relation to its absolute Gross Domestic Product in US Dollars.

In striking contrast to the overall increase of caesarean section numbers over the past decades, there seem to be large differences in the indication for caesarean sections between countries and even single regions. Specifically, the global profile of caesarean section rates has been addressed by Gibbons *et al.* in a World Health report background paper issued by the World Health Organization (WHO) [31]. Their study estimated country-specific section rates and presented data ranging from low-income countries such as the Chad with a rate of 0.4% caesarean sections per all annual births to Brazil and the Dominican Republic with rates of 45.9% and 41.9%, respectively. When we compared the research activity to the overall numbers of caesarean sections as published by the WHO World Health report background paper, we found interesting differences concerning the relation of research activities and caesarean section numbers: Countries with extremely high absolute numbers of caesarean section procedures such as the USA or Germany drop out of the rankings. On the contrary, Scandinavian countries such as Sweden or Finland showed a high relative research activity in the field of caesarean section research. These differences cannot simply be explained by how many caesarean sections were performed in these countries since they do not have higher caesarean section rates compared to other high income countries [31]. On the contrary, Sweden, Norway and Finland all have relatively low procedure rates (17.3%, 16.6% and 16.3% caesarean sections per annual births, respectively). We hypothesize that it might be a priority for these countries to conduct research in this important field, which impacts the health and life of many women and children. Additionally, Scandinavian countries ranked among the top nations when country-specific citation rates were investigated: Sweden had the highest citation rate with 20.4 citations per caesarean section publication followed by Norway and Finland with citation rates of 16.3 each. This finding might be explained by large epidemiological databases that are established in Scandinavian countries, e.g. the Swedish and Danish Birth Registers or Swedish National Patient Register. These vast data resources enable researchers to conduct key studies, which are extremely relevant for the field, of exceptional quality and lead to many citations.

With regard to the overall dominating role of the USA concerning the research activity, it is crucial to relate the publication output to benchmarks of socioeconomic welfare since rich countries should be obliged to invest more resources in research than low-income countries. The relation, *i.e.*, of caesarean section research activity to a relative prosperity index such as the GDP *per capita*, shows that the USA still has a leading position in this ranking. However, when the caesarean section research activity is related to absolute benchmarks of economic well-being such as the absolute GDP in current USD, it is found that the USA lost its primary position and was ranked 15th. By contrast, the UK, which is ranked 2nd in absolute research activity, stayed at a high position with rank 3, indicating that this country has a high relative economic investment concerning caesarean section research. Further, we could deduce from our data that countries with a very high caesarean section procedure rate such as Brazil or Italy and Mexico invest rather low research resources into this area, although it should be anticipated that countries with high caesarean section numbers would dedicate larger resources to conduct caesarean section-related research. We hypothesize that a country would do so because it feels obligated to serve the health of its population, and the research could rely on an abundance of epidemiological data. Such scientific endeavors should target areas like the prevention of complications associated with caesarean sections, the implementation of evidence-based strategies for avoiding medically unnecessary caesarean sections, and the safe and appropriate use of vaginal birth after a caesarean section [33]. We also found that caesarean section numbers are low in many low- and middle-income countries, which might be explained with a limited access to surgical procedures or a different cultural attitude towards this type of delivery in these countries. To the same extent, we documented a very limited research activity in relation to procedure numbers or economic strength in these countries as illustrated by density equalizing procedures.

When the presently established picture of the global caesarean section research activity is compared to other fields of obstetrics and gynecology, two other studies may be used for comparison: a study by on smoking and pregnancy [34] and a study on breast cancer research activities [35]. Concerning smoking and pregnancy, Mund *et al.* reported that out of 10,043 publications related to smoking and pregnancy, the highest number of scientific papers was published in the USA (35.5%), followed by the UK (9.9%) and Canada (5.3%) [34]. These nations also achieved the highest modified h-Indices of 128, 79 and 62, which was congruent to our results, and the highest citation rates of 41.4%, 8.6% and 5.3%, respectively [34]. With regard to breast cancer research, a similar approach as ours was used [35]: Data were retrieved from the Web of Science database using the Boolean operator, “OR”, with different terms related to breast cancer, including “breast cancer”, “mammary ductal carcinoma” and “breast tumor”. In contrast to the currently limited amount of caesarean sections publications, a total of 180,126 breast cancer-associated items were produced over the study period from 1954–2008 [35]. As in our study, the USA issued the highest output (*n* = 77,101) and was followed by the UK (*n* = 18,357) and Germany (*n* = 12,529). Regarding international collaborative research, the findings by Glynn *et al.* mirror our results on caesarean section-related research: The authors described that collaborative scientific activity on breast cancer continued to increase annually since publication of the first collaborative article in 1973. A peak was reached in 2008, with 3127 items. In their analysis, bilateral cooperations were also the most common with the majority established between the USA and Canada followed by collaborations between the USA and the UK [35].

We need to address the following methodological issues: The Web of Science was used as an underlying database for the acquisition of raw data due to its ability to generate detailed information regarding citation characteristics of single database entries. We were therefore able to construct so-called semi-qualitative indices such as country-specific citation rates or h-Indices, which allowed us to integrate these valuable aspects into the study. As in every database, we encountered language biases using the WoS since many national, non-English written OB/GYN journals are not enlisted or underrepresented in this resource. This aligns with our finding that 93% of identified publications were authored in English. Hence, we can assume that the publication activity of the USA, the UK and other English speaking countries might be slightly overestimated in our analysis.

The procedure numbers for caesarean sections are worldwide on the rise. This development is becoming a major public health concern and causes discussions regarding costs, associated perinatal mortality and morbidity as well as inequity in access [36,37,38]. Hence, studies have to be conducted to re-address the questions for the ideal caesarean section rate in a population. We know that this “baseline” caesarean section rate is hard to define. It should guarantee enough access to this surgical procedure to ensure favorable outcomes for as many mothers and children as possible and should not contribute unnecessarily to perinatal morbidity and mortality at the same time. Our study helps to shed light on the worldwide ongoing caesarean section research activities and existing collaborative networks. Our data showed the need of high-income countries with high caesarean delivery rates to conduct even more caesarean section-related research. They should investigate the particular reasons for the increasing caesarean section rates and explore ways to limit their numbers of surgical deliveries. On the other hand, low-income countries should be included in collaborative research efforts or receive funding to address the present healthcare disparity.

## 4. Conclusions

Caesarean section is a common surgical life saving procedure both for the mother and the child and of high importance for the reduction of perinatal mortality. We here depicted a first sketch of the global caesarean section research architecture and analyzed quantitative and semi-qualitative aspects of the research over a period of more than 100 years. As in many other fields of medicine, the USA dominated most parameters. Interestingly, countries with the highest caesarean section numbers such as China or Brazil did not play prominent roles in the research activity rankings. As with other research fields in obstetrics and gynecology, caesarean section research activity also follows the global health care inequity pattern since it is underpowered in low-income settings. Therefore, allocation of research funding may be directed into countries with difficult access to this important obstetrical procedure aiming to address an important healthcare disparity.
